# Cationic Surfactant-Based Colorimetric Detection of *Plasmodium* Lactate Dehydrogenase, a Biomarker for Malaria, Using the Specific DNA Aptamer

**DOI:** 10.1371/journal.pone.0100847

**Published:** 2014-07-03

**Authors:** Seonghwan Lee, D H Manjunatha, Weejeong Jeon, Changill Ban

**Affiliations:** Department of Chemistry, Pohang University of Science and Technology, Pohang, Gyeongbuk, Republic of Korea; Royal Tropical Institute, Netherlands

## Abstract

A simple, sensitive, and selective colorimetric biosensor for the detection of the malarial biomarkers *Plasmodium vivax* lactate dehydrogenase (*Pv*LDH) and *Plasmodium falciparum* LDH (*Pf*LDH) was demonstrated using the pL1 aptamer as the recognition element and gold nanoparticles (AuNPs) as probes. The proposed method is based on the aggregation of AuNPs using hexadecyltrimethylammonium bromide (CTAB). The AuNPs exhibited a sensitive color change from red to blue, which could be seen directly with the naked eye and was monitored using UV-visible absorption spectroscopy and transmission electron microscopy (TEM). The reaction conditions were optimized to obtain the maximum color intensity. *Pv*LDH and *Pf*LDH were discernible with a detection limit of 1.25 pM and 2.94 pM, respectively. The applicability of the proposed biosensor was also examined in commercially available human serum.

## Introduction

Malaria is a life-threatening parasitic disease, which involves high fevers, shaking chills, flu-like symptoms, and anemia, and is transmitted to people through the bites of infected mosquitoes. According to the World Health Organization (WHO), in 2012, there were about 207 million cases of malaria globally (with an uncertainty range of 135 million to 287 million) and an estimated 627,000 deaths (with an uncertainty range of 473,000 to 789,000), mostly among African children [1]. Hence, the diagnosis and treatment of malaria is very important for the welfare of mankind. According to the literature, there are few methods available for the diagnosis of malaria. Malaria is commonly diagnosed based on parasitological confirmation by either microscopy or rapid diagnostic tests (RDTs) [2]. However, each of these methods has several limitations. The microscopy method needs a highly trained, experienced expert, and a tedious procedure is followed for the preparation of blood samples. Even though RDTs are commercially available, they have inadequate sensitivity and specificity at low parasite concentrations. Moreover, RDT is vulnerable to heat and humidity, because RDT is based on an antibody-antigen recognition. Due to these limitations, a rapid and accurate method for the diagnosis of malaria is essential.

Aptamers are oligonucleic acid or peptide molecules that bind to specific target molecules; hence, aptamers are considered to be alternatives to antibodies in both therapeutic and diagnostic applications [3]–[6]. Aptamers have many advantages, including their ease of discovery and analysis, specificity to targets, thermal stability, and convenience of chemical synthesis [7]–[13]. Thus, research into the development of aptasensors has drawn significant attention from scientists and is being studied intensively. In particular, an aptasensor for detection of malaria is required to overcome the drawbacks of current diagnostic methods. In our previous work, we reported the development of aptamers for the malaria biomarkers, *Plasmodium vivax* LDH (*Pv*LDH) and *Plasmodium falciparum* LDH (*Pf*LDH), through the systematic evolution of ligands by exponential enrichment (SELEX) [14].

Colorimetry has commonly been used for routine analysis since it does not require any special instruments and can be observed directly with the naked eye. Gold nanoparticle (AuNP)-based colorimetric assays have recently drawn extensive attention for their simplicity, ease of observation, and the lack of need for complicated instrumentation [15], [16]. Since the introduction of the first DNA sensor based on mercaptoalkyloligonucleotide-modified AuNPs and a target single-stranded DNA (ssDNA) through hybridization [17], similar platforms, such as DNA, proteins, heavy metal ions, and small molecules, have been developed to analyze various substances [18]–[21]. Most of the assays are based on target-mediated crosslinking mechanisms through the modification of AuNPs with specific binding-ligands [22], [23]. In practice, however, assay procedures based on the non-crosslinking aggregation mechanism of AuNPs are more convenient and cost-effective due to the absence of the elaborate and expensive synthesis of thiol-containing ligand-modified AuNPs [24], [25]. The loss of steric stabilization in DNA-stabilized AuNPs is one of the main approaches used to induce colloid aggregation through a non-crosslinking mechanism. This can be achieved by changing the DNA structure, e.g., forming a double-stranded DNA (dsDNA), or through the loss of DNA stabilizers, e.g., enzymatic cleavage of DNA [26].

Recent research has demonstrated that hexadecyltrimethylammonium bromide (CTAB), a cationic surfactant, can be used to prepare AuNPs in controllable sizes and shapes [27]–[29]. CTAB displays two useful features: a positive charge, which can be employed to aggregate AuNPs, and the ability to assemble DNA to form supramolecules with certain nanostructures [30]–[32]. Some studies have shown that long-chain, single-stranded DNA forms cubic nanostructures with CTAB, whereas long-chain dsDNA forms hexagonal nanostructures [33]. Recently, Wu *et al*., developed a colorimetric biosensor for the detection of metal ions using CTAB [34], but so far the proposed strategy has not been applied to the detection of a protein.

In this work, we provide the demonstration of the sensitive and selective colorimetric detection of recombinant *Pv*LDH and *Pf*LDH proteins using the pL1 aptamer [14] as the recognition element and AuNPs as probes. CTAB was considered an efficient material to aggregate AuNPs. The useful property of CTAB is that, it not only aggregates the AuNPs, but also controls their aggregation via its competitive binding to aptamers [34]. The colorimetric aptasensor is a rapid, accurate, sensitive, and specific biosensor for the detection of *Plasmodium* LDH (pLDH) proteins. In addition, the proposed biosensor is applied to the detection of *Pv*LDH and *Pf*LDH spiked with human serum.

## Experimental

### Materials and instruments

The pL1 aptamer (5′-GTT CGA TTG GAT TGT GCC GGA AGT GCT GGC TCG AAC-3′) was synthesized by Bionics (Korea), and the BL21(DE3) *Escherichia coli* strain was purchased from Invitrogen (USA). The CTAB, the human serum (product number: H4522), the albumin from human serum (HAS), the γ-globulins from human blood, and the fibrinogen from human plasma were purchased from Sigma-Aldrich (USA). The Hi-trap Ni-NTA affinity column, MonoQ anion exchange column, and Superdex 200 HL gel filtration column were purchased from GE Healthcare (USA). The AKTA Purifier Fast Protein Liquid Chromatography system was used for the purification of the *Pv*LDH, *Pf*LDH, and the human LDH A chain (hLDHA). The absorbance measurements were performed using the HP 845x UV-Visible System.

### Preparation of LDH proteins

The *Pv*LDH and *Pf*LDH proteins were obtained using a bacterial expression system in accordance with the literature [14], [35]. Briefly, the competent cells containing the *Pv*LDH gene were grown at 37°C for 4 h and lysed by sonication at 4°C in a lysis buffer (20 mM Tris-HCl, pH 8.0, 500 mM NaCl, 0.5 mM β-mercaptoethanol, 10 mM imidazole, and 5% [w/v] glycerol). The proteins were purified using the Hi-trap Ni-NTA affinity column, the MonoQ anion exchange column and the Superdex 200 HL gel filtration column. The purified proteins were stored in a storage buffer (50 mM HEPES, pH 7.4, 5 mM β-mercaptoethanol, and 5% [w/v] glycerol) at −80°C. The *Pf*LDH proteins and hLDHA were obtained using the same procedure. The purity of each protein was analyzed using sodium dodecylsulfate polyacrylamide gel electrophoresis (SDS-PAGE).

### Preparation of the gold nanoparticles

In accordance with the literature [36], the AuNPs were synthesized with a citrate reduction of HAuCl_4_ in which trisodium citrate was employed as a stabilizer capped on the AuNPs surface. All glassware was treated with aqua regia, which was prepared using freshly mixed, concentrated nitric acid and hydrochloric acid in a volume ratio of 1∶3. A 1.0 mM HAuCl_4_ solution (100 mL) was boiled and stirred vigorously, and a 38.8 mM trisodium citrate solution (10 mL) was then added rapidly. The mixture boiled for 30 min until a wine-red color was obtained and then cooled to room temperature. Next, it was filtered through a 0.22 µm membrane filter to remove the precipitate, and the filtrate was stored in a refrigerator at 4°C for future use. The concentration of AuNPs was measured by UV-visible absorption spectroscopy, and the AuNPs were concentrated to 10 nM. The size of the AuNPs was confirmed using transmission electron microscopy (TEM).

### Optimization of detection conditions

Various concentrations of CTAB (0, 1, 1.5, 2, 2.5, 3, 3.5, 4, 4.5, and 5 nM) in a reaction buffer (160 µL, 20 mM HEPES, and pH 7.4) were added to the AuNP solution (40 µL) to optimize the detection conditions. Absorbance values at 520 nm and 650 nm were recorded by the UV-visible absorption spectrometer. Similarly, a suitable concentration of the pL1 aptamer was investigated for the optimization of detection conditions. Various concentrations of the pL1 aptamer (0, 1, 2, 5, 10, 20, 50, and 100 nM) and 4 nM of CTAB were incubated in an identical volume of the reaction buffer for 20 min at room temperature. Next, the AuNP solution was added to the mixture and the absorbance values at 520 nm and 650 nm were measured. In order to optimize the concentration of CTAB for detection in the human serum sample, various concentrations of CTAB (0, 1, 5, 10, 30, 50, 100, 200, and 300 nM), 20 nM of the pL1 aptamer, and a prepared human serum sample (4 µL) were incubated in the reaction buffer (160 µL) for 20 min at room temperature. After the AuNP solution was mixed with the reaction buffer, the change in the absorbance values was measured using the UV-visible absorption spectrometer.

### Colorimetric detection of recombinant pLDH

An appropriate volume of 20 nM of the aptamer solution and varying concentrations of the recombinant pLDH were mixed thoroughly in the reaction buffer, and then incubated for 20 min at room temperature. Subsequently, 4 nM of CTAB was added to the mixed solutions and incubated for 20 min at room temperature. Finally, the AuNP stock solution was added to provide a final volume of 200 µL. After incubation for 5 min at room temperature, the absorbance values at 520 nm and 650 nm were measured. The experiments were carried out separately three times. The ratio of A650/A520 of each sample solution and absorbance spectra was obtained using the UV-visible absorption spectrometer, and the AuNP color changes were observed with the naked eye.

### Detection of pLDH in the human serum sample

The detection of recombinant pLDH proteins in the human serum sample was performed using an identical protocol. Several serum samples were prepared by adding various concentrations of pLDH into the commercially available human serum. The serum samples were diluted 10 times with phosphate buffered saline (PBS). 4 µL of pretreated serum samples and 20 nM of the pL1 aptamer were added to the reaction buffer solution, and the mixture solutions were incubated for 20 min at room temperature. Thereafter, 100 nM of CTAB was added to the mixtures, and they were incubated for 20 min at room temperature. The AuNP solutions were then mixed in each sample, and the absorbance values were recorded. Moreover, the dispersed and aggregated AuNPs were confirmed using TEM.

## Results and Discussion

### Principle of colorimetric detection for pLDH

In this work, the synthesized AuNP solution (Fig. S1) was stabilized by the citrate anions as their repulsion prevented the AuNPs from aggregating. Electrosteric stabilization is a major factor for ensuring that AuNPs remain dispersed in an aqueous solution. Generally, positively charged materials, such as cationic polymers, surfactants, and high concentrations of sodium, can disturb the charge balance of AuNPs and cause them to aggregate.

**Figure 1:**
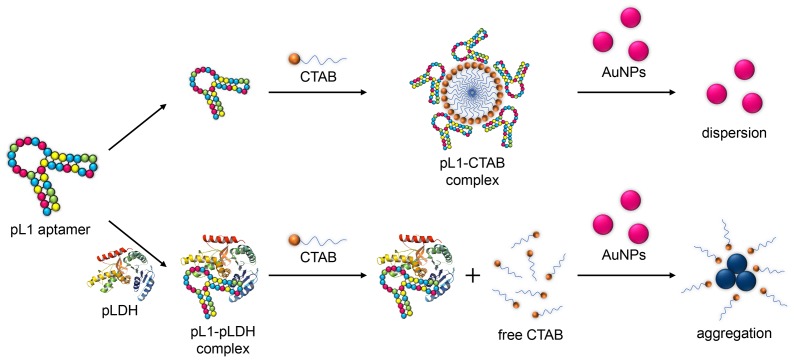
Schematic representation of the biosensor. Schematic representation of the biosensor for the detection of pLDH based on the surfactant-induced aggregation of AuNPs.

In this study, CTAB was used to aggregate AuNPs, and the pL1 aptamer played two major roles: (i) it interacted with pLDH to form an aptamer-protein complex; and (ii) it assembled with the CTAB to make supramolecules, which prevented the AuNPs from aggregating. The mechanism of the pLDH biosensor is shown in Fig. 1. In the absence of pLDH, the pL1 aptamers were free and could assemble with the CTAB to form supramolecules, and thus the subsequent AuNPs could not aggregate due to the lack of free CTAB. On adding pLDH, the pL1 aptamer could specifically bind to the pLDH and induce the pL1 aptamer-pLDH complex. Thus, the pL1 aptamers were exhausted due to the formation of the aptamer-pLDH complex, allowing the free CTAB to aggregate the AuNPs, which led to the remarkable signal change in absorbance. The signal variation of the solution depended on the concentration of free CTAB, which was in turn directly conditioned by the concentration of pLDH. Therefore, this strategy makes it possible to detect pLDH via the colorimetric method. The results confirm that the color change was correlated with the degree of AuNP aggregation caused by the interaction of the AuNPs and CTAB, depending on the concentration of pLDH.

### Optimization of detection conditions

Optimal reaction conditions for the effective quantitative determination of pLDH were established through the performance of several experimental trials. It was observed that the sensitivity of the method depends on the concentration of CTAB and the pL1 aptamer.

**Figure 2:**
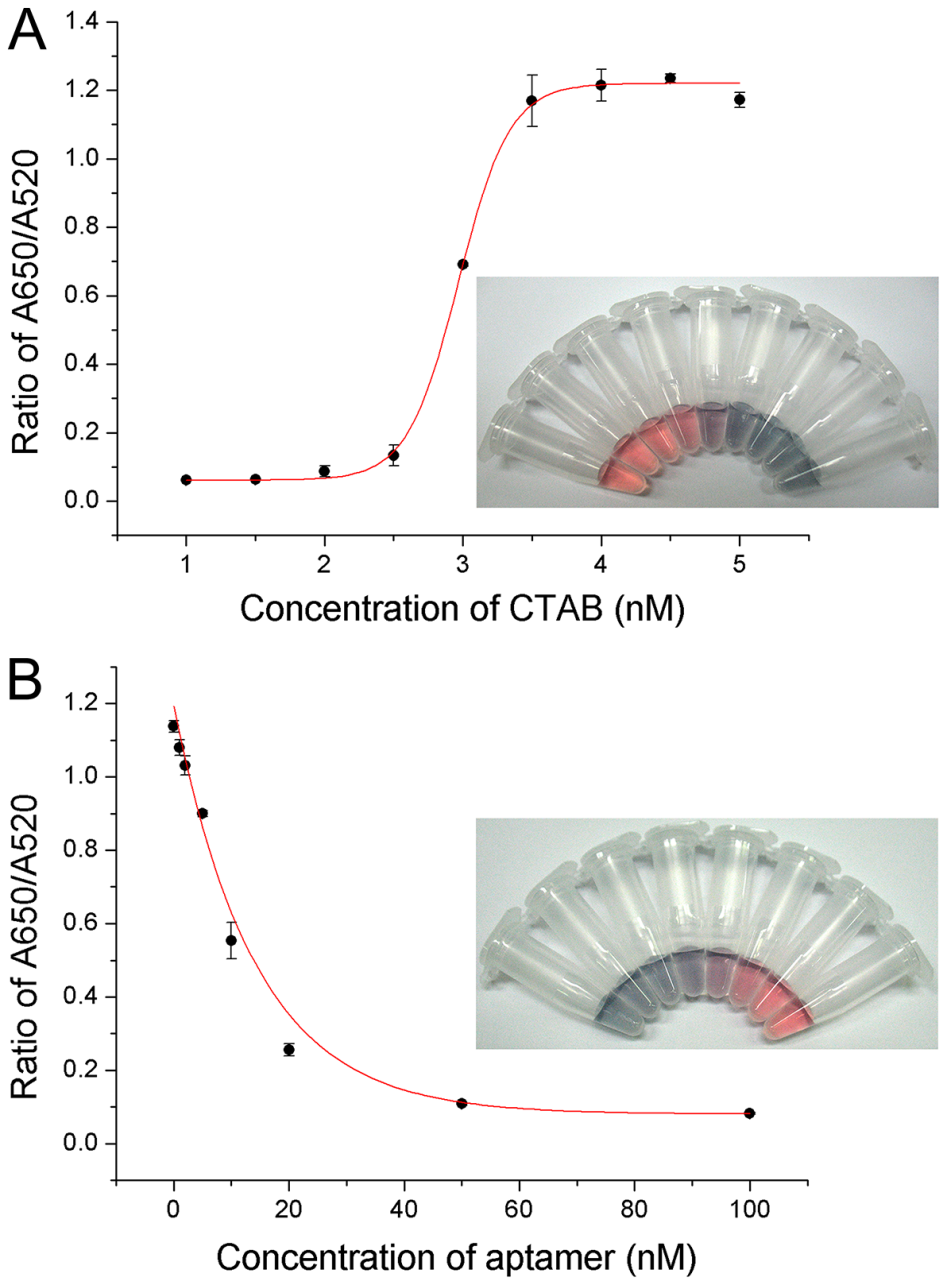
Optimization of CTAB and pL1 aptamer concentrations. (A) The effect of the concentration of CTAB on pLDH detection. The inset figure represents the visual color change of the AuNPs at each concentration level. (B) The effect of the concentration of the pL1 aptamer on pLDH detection. The inset figure shows the visual color change of the AuNPs at each concentration level. (A and B) Points and error bars represents the means and standard deviations, respectively, of three repeated measurements.

To optimize the concentration of CTAB required for the aggregation of the AuNPs, varying concentrations of CTAB (0–5 nM) were added to the reaction buffer solution, followed by a constant concentration of AuNP solution (10 nM). The absorbance values at 520 nm and 650 nm were recorded, and the ratio of A650/A520 was calculated. The color of the AuNP solutions changed gradually from wine red to purple and blue (Fig. 2A). The values of A650/A520 ratios increased until the concentration of CTAB reached 4 nM. At this point, all AuNPs aggregated thoroughly. With a further increase in the CTAB concentration, there was no significant increase in the values. The reason for this phenomenon was that the AuNPs had aggregated thoroughly to form large particles at a high concentration of CTAB. Thus, 4 nM of CTAB was considered to be the proper concentration for the colorimetric aptasensor.

To identify the best concentration of the pL1 aptamer, similar experiments were carried out with varied concentrations of the aptamer (0–100 nM) in the reaction buffer solution containing the fixed concentration of CTAB (4 nM). The ratio of A650/A520 was decreased until 20 nM of the pL1 aptamer was reached, after which it did not decrease considerably (Fig. 2B). Therefore, 20 nM of the pL1 aptamer was considered suitable for the effective detection of pLDH.

### Colorimetric detection of recombinant *Pv*LDH and *Pf*LDH

**Figure 3:**
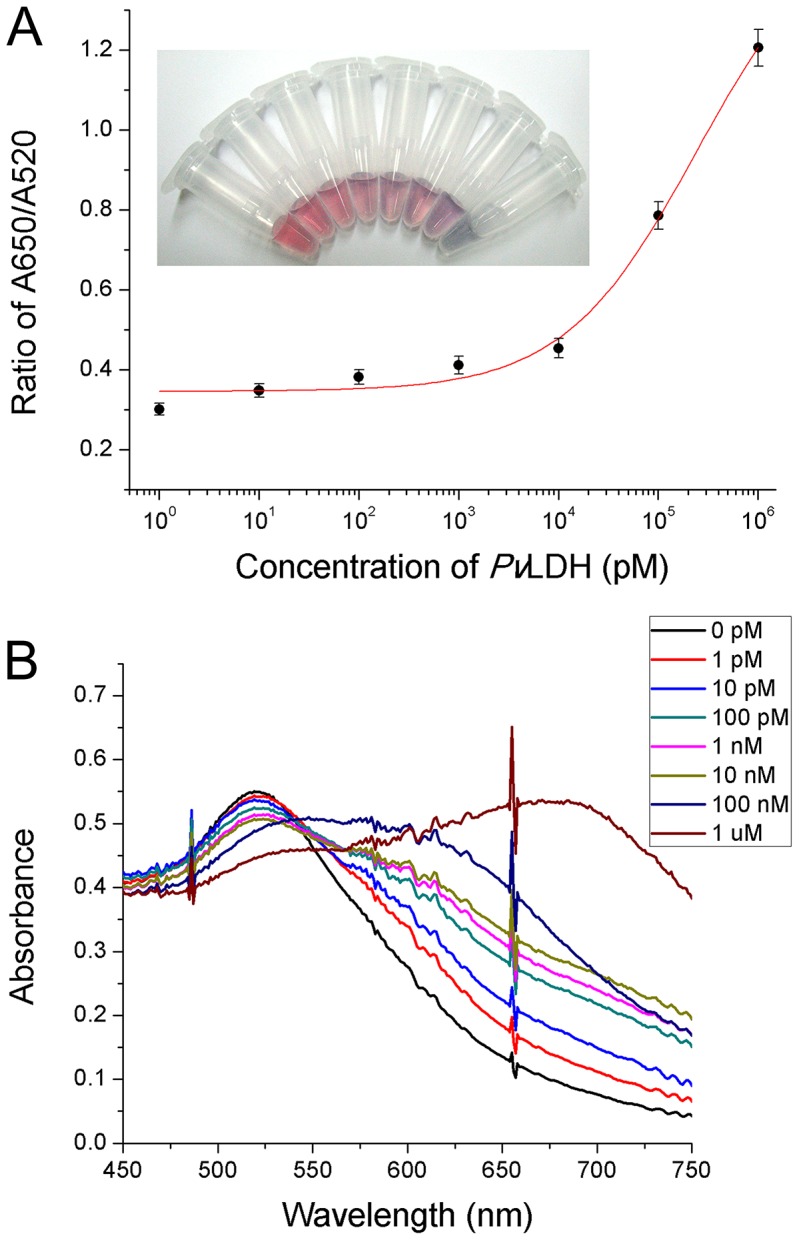
Colorimetric detection of recombinant *Pv*LDH. (A) The calibration curve of the sensing solutions containing varying concentrations of *Pv*LDH. Points and error bars represents the means and standard deviations, respectively, of three repeated measurements. The inset figures represent the visual color changes of the AuNP solutions. (B) The absorbance spectra for the detection of *Pv*LDH.

Given the optimized detection conditions, detection of the recombinant *Pv*LDH was performed with the naked eye and the UV-visible absorption spectrometer. In the absence of *Pv*LDH, there was no free CTAB in the reaction buffer because the pL1 aptamer and CTAB had formed supramolecules. Hence, the AuNPs in the reaction buffer were dispersed, and its color remained red. In the present of various concentrations of *Pv*LDH (0 pM, 1 pM, 10 pM, 100 pM, 1 nM, 10 nM, 100 nM, and 1 µM), the amount of free CTAB gradually increased as the concentration of *Pv*LDH increased because the pL1 aptamer bound more strongly to the *Pv*LDH protein compared to the CTAB. This led to the aggregation of the AuNPs to the extent that the color of the AuNP solution changed dramatically from red to purple (Fig. 3A). Moreover, the absorbance spectra were obtained for further confirmation, and a blue shift of AuNPs was observed (Fig. 3B). The absorbance ratio of A650/A520 was increased depending on the increase in the concentration of *Pv*LDH. The results showed that the absorption ratio of A650/A520 was linear over the range of 1 pM to 10 nM, and the calculated detection limit was 1.25 pM. In addition, experiments with *Pf*LDH were carried out using the identical protocol, and a detection limit of 2.94 pM was obtained (Fig. S2). These values indicated that the proposed biosensor was highly sensitive. The results further revealed that a high concentration of pLDH prevented the formation of a pL1 aptamer-CTAB complex and induced the existence of free CTAB, which in turn aggregated the AuNPs. Thus, the pLDH proteins were successfully detected by the colorimetric aptasensor.

### Selectivity of the biosensor

The selectivity of the aptasensor towards the pLDH was investigated using several other human proteins, including HSA, γ-globulins, fibrinogen, and hLDHA. To evaluate the selectivity of the biosensor, the same protocol was used, and the concentrations of the pL1 aptamer, CTAB, and AuNPs were fixed as for detection of pLDH. Each protein (1 µM) was sequentially incubated with the pL1 aptamer, CTAB, and AuNPs, and then the absorbance ratio of A650/A520 was recorded. The results revealed that the A650/A520 values of *Pv*LDH and *Pf*LDH increased significantly whereas those of the other proteins did not increase compared with the control sample (Fig. S3). These results demonstrate that the proposed biosensor is highly selective towards recombinant *Pv*LDH and *Pf*LDH proteins.

### Detection of pLDH in the human serum sample

The detection of pLDH proteins in the human serum sample is critical for the utilization of the aptasensor as a diagnostic tool for malaria. Therefore, an examination of the aptasensor performance in the human serum sample was essential. The optimization of the detection conditions for the human serum sample was carried out to adjust the sensing conditions to the complex biomolecular environment of the human serum. These biomolecules are charged, so the formation of the aptamer-protein complex or aptamer-surfactant complex could be affected, which could lead to effective detection failure. Moreover, various enzymes could cause an enzymatic reaction with probe molecules or AuNPs, and these reactions could interfere with colorimetric detection. For these reasons, detection condition for the human serum sample must be optimized.

In order to determine suitable concentrations of CTAB for detection in the human serum sample, various concentrations of CTAB were tested with the commercially available human serum sample using similar protocols as for the optimization of detection. The result revealed that 100 nM of CTAB was the appropriate concentration to detect pLDH proteins in the human serum sample (Fig. S4).

**Figure 4:**
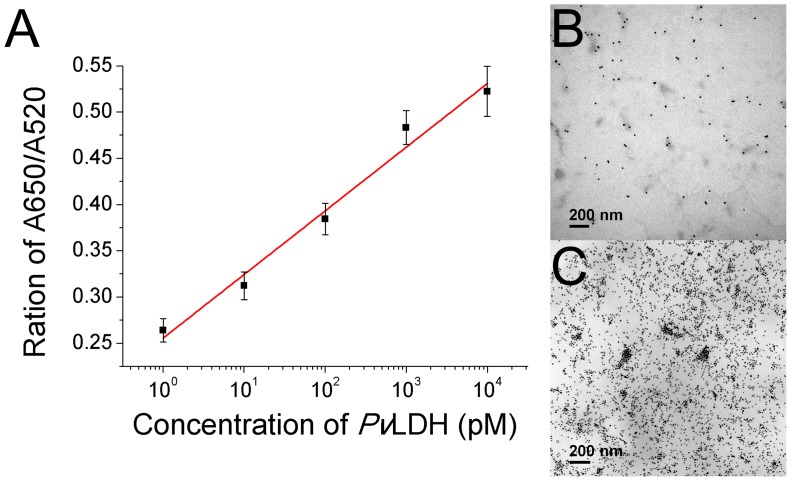
Detection of *Pv*LDH in the human serum sample. (A) The calibration curve of the sensing solutions containing varying concentrations of *Pv*LDH in the serum sample. Points and error bars represents the means and standard deviations, respectively, of three repeated measurements. (B) The TEM image of the AuNP solution containing the serum sample. (C) The TEM image of the AuNP solution containing 10 nM of *Pv*LDH in the serum sample.

Detection of pLDH in the human serum sample was performed for both the *Pv*LDH and *Pf*LDH to investigate the probability of success in a diagnostic application. Each serum sample was prepared by adding varying concentration of pLDH proteins (0 pM, 1 pM, 10 pM, 100 pM, 1 nM, and 10 nM). The absorbance values at 520 nm and 650 nm were measured, and the ratio of A650/A520 increased in parallel with the increase in protein concentration (Fig. 4A and Fig. S5). The detection limits of both *Pv*LDH and *Pf*LDH were calculated using the linear fit, and the values were 10.17 pM and 13.54 pM, respectively. The aggregation of the AuNPs induced by the detection of pLDH in the human serum sample was further verified by TEM imaging. In the case of the absence of *Pv*LDH, the AuNPs were dispersed (Fig. 4B). However, the AuNPs in the reaction solution containing the *Pv*LDH (10 nM) were highly aggregated due to free CTAB, which existed in the solution due to the formation of the pL1 aptamer-*Pv*LDH complex (Fig. 4C). These results suggest that the aptasensor can be utilized to detect pLDH in the human serum. There are still further steps that are required to validate the usefulness of the aptasensor, but it has been verified that the proposed aptasensor has the potential to become a diagnostic tool for malaria.

## Conclusion

In summary, CTAB-based label-free colorimetric sensing of pLDH was performed with the pL1 aptamer and AuNPs. The proposed biosensor offers simple, rapid, and sensitive detection of pLDH proteins. In comparison with other pLDH sensors, the present method can rapidly detect low concentrations of pLDH through observation with the naked eye and UV-visible absorption spectroscopy. Both *Pv*LDH and *Pf*LDH were selectively detected using the aptasensor and showed low detection limits of 1.25 pM for *Pv*LDH and 2.94 pM for *Pf*LDH, whereas the aptasensor did not work for other proteins, such as HSA, γ-globulins, fibrinogen, and hLDHA. In addition, detection of pLDH in the human serum sample was performed using UV-visible spectroscopy and TEM imaging. Both *Pv*LDH and *Pf*LDH in serum samples were successfully detected with low detection limits. Therefore, the proposed aptasensor for pLDH can be utilized for the accurate and sensitive detection of pLDH in the human serum.

## Supporting Information

Figure S1 TEM image of the synthesized AuNPs. The AuNPs were synthesized with a citrate reduction of HAuCl_4_ and their size confirmed using TEM.
(DOCX)

Figure S2 Colorimetric detection of recombinant *Pf*LDH. (A) The calibration curve of the sensing solutions containing varying concentrations of *Pf*LDH. Points and error bars represents the means and standard deviations, respectively, of three repeated measurements. The inset figures represent the visual color changes of the AuNP solutions. (B) The absorbance spectra for the detection of *Pf*LDH.
(DOCX)

Figure S3 The selectivity of the aptasensor. The selectivity of the aptasensor for the pLDH proteins. The values of A650/A520 of each competing protein were measured with the same concentration of 1 µM.
(DOCX)

Figure S4 Optimization of CTAB concentration for detection in serum samples. The effect of the concentration of CTAB on pLDH detection in serum sample. Points and error bars represents the means and standard deviations, respectively, of three repeated measurements.
(DOCX)

Figure S5 Detection of *Pf*LDH in the human serum sample. The calibration curve of the sensing solutions containing varying concentrations of *Pf*LDH in the serum sample. Points and error bars represents the means and standard deviations, respectively, of three repeated measurements.
(DOCX)

## References

[1] World Health Organization (2012) World malaria report. Geneva, Switzerland: World Health Organization.

[2] Maltha J, Gamboa D, Bendezu J, Sanchez L, Cnops L, et al (2012) Rapid diagnostic tests for malaria diagnosis in the Peruvian Amazon: impact of pfhrp2 gene deletions and cross-reactions. PLoS One 7: e43094. 22952633 10.1371/journal.pone.0043094 PMC3429466

[3] Jayasena SD (1999) Aptamers: an emerging class of molecules that rival antibodies in diagnostics. Clin Chem 45: 1628–1650. 10471678

[4] Song KM, Lee S, Ban C (2012) Aptamers and Their Biological Applications. Sensors 12: 612–631. 22368488 10.3390/s120100612 PMC3279232

[5] Min K, Jo H, Song K, Cho M, Chun YS, et al (2011) Dual-aptamer-based delivery vehicle of doxorubicin to both PSMA (+) and PSMA (−) prostate cancers. Biomaterials 32: 2124–2132. 21147500 10.1016/j.biomaterials.2010.11.035

[6] Min K, Song KM, Cho M, Chun YS, Shim YB, et al (2010) Simultaneous electrochemical detection of both PSMA (+) and PSMA (−) prostate cancer cells using an RNA/peptide dual-aptamer probe. Chemical Communications 46: 5566–5568. 20407731 10.1039/c002524k

[7] Jiang Y, Zhao H, Lin Y, Zhu N, Ma Y, et al (2010) Colorimetric detection of glucose in rat brain using gold nanoparticles. Angew Chem Int Ed Engl 49: 4800–4804. 20533481 10.1002/anie.201001057

[8] Centi S, Tombelli S, Minunni M, Mascini M (2007) Aptamer-based detection of plasma proteins by an electrochemical assay coupled to magnetic beads. Anal Chem 79: 1466–1473. 17297945 10.1021/ac061879p

[9] Medley CD, Smith JE, Tang Z, Wu Y, Bamrungsap S, et al (2008) Gold nanoparticle-based colorimetric assay for the direct detection of cancerous cells. Anal Chem 80: 1067–1072. 18198894 10.1021/ac702037y

[10] Maehashi K, Katsura T, Kerman K, Takamura Y, Matsumoto K, et al (2007) Label-free protein biosensor based on aptamer-modified carbon nanotube field-effect transistors. Anal Chem 79: 782–787. 17222052 10.1021/ac060830g

[11] Song KM, Cho M, Jo H, Min K, Jeon SH, et al (2011) Gold nanoparticle-based colorimetric detection of kanamycin using a DNA aptamer. Anal Biochem 415: 175–181. 21530479 10.1016/j.ab.2011.04.007

[12] Wang JL, Munir A, Zhou HS (2009) Au NPs-aptamer conjugates as a powerful competitive reagent for ultrasensitive detection of small molecules by surface plasmon resonance spectroscopy. Talanta 79: 72–76. 19376346 10.1016/j.talanta.2009.03.003

[13] Hayat A, Sassolas A, Marty JL, Radi A (2013) Highly sensitive ochratoxin A impedimetric aptasensor based on the immobilization of azido-aptamer onto electrografted binary film via click chemistry. Talanta 103: 14–19. 23200352 10.1016/j.talanta.2012.09.048

[14] Lee S, Song KM, Jeon W, Jo H, Shim YB, et al (2012) A highly sensitive aptasensor towards Plasmodium lactate dehydrogenase for the diagnosis of malaria. Biosens Bioelectron 35: 291–296. 22459583 10.1016/j.bios.2012.03.003

[15] Rosi NL, Mirkin CA (2005) Nanostructures in biodiagnostics. Chem Rev 105: 1547–1562. 15826019 10.1021/cr030067f

[16] Zhang LP, Hu B, Wang JH (2012) Label-free colorimetric sensing of ascorbic acid based on Fenton reaction with unmodified gold nanoparticle probes and multiple molecular logic gates. Anal Chim Acta 717: 127–133. 22304824 10.1016/j.aca.2011.12.037

[17] Elghanian R, Storhoff JJ, Mucic RC, Letsinger RL, Mirkin CA (1997) Selective colorimetric detection of polynucleotides based on the distance-dependent optical properties of gold nanoparticles. Science 277: 1078–1081. 9262471 10.1126/science.277.5329.1078

[18] Cho M, Han MS, Ban C (2008) Detection of mismatched DNAs via the binding affinity of MutS using a gold nanoparticle-based competitive colorimetric method. Chem Commun (Camb): 4573–4575. 10.1039/b811346g 18815687

[19] Lin JH, Chang CW, Wu ZH, Tseng WL (2010) Colorimetric Assay for S-Adenosylhomocysteine Hydrolase Activity and Inhibition Using Fluorosurfactant-Capped Gold Nanoparticles. Anal Chem 82: 8775–8779. 20945873 10.1021/ac102020n

[20] Lin CY, Yu CJ, Lin YH, Tseng WL (2010) Colorimetric sensing of silver(I) and mercury(II) ions based on an assembly of Tween 20-stabilized gold nanoparticles. Anal Chem 82: 6830–6837. 20704372 10.1021/ac1007909

[21] Kim YS, Kim JH, Kim IA, Lee SJ, Jurng J, et al (2010) A novel colorimetric aptasensor using gold nanoparticle for a highly sensitive and specific detection of oxytetracycline. Biosens Bioelectron 26: 1644–1649. 20829027 10.1016/j.bios.2010.08.046

[22] Stewart ME, Anderton CR, Thompson LB, Maria J, Gray SK, et al (2008) Nanostructured plasmonic sensors. Chem Rev 108: 494–521. 18229956 10.1021/cr068126n

[23] Cho M, Han MS, Ban C (2008) Detection of mismatched DNAs via the binding affinity of MutS using a gold nanoparticle-based competitive colorimetric method. Chemical Communications: 4573–4575. 10.1039/b811346g 18815687

[24] Sato K, Hosokawa K, Maeda M (2003) Rapid aggregation of gold nanoparticles induced by non-cross-linking DNA hybridization. J Am Chem Soc 125: 8102–8103. 12837070 10.1021/ja034876s

[25] Sato K, Hosokawa K, Maeda M (2005) Non-cross-linking gold nanoparticle aggregation as a detection method for single-base substitutions. Nucleic Acids Res 33: e4. 15640441 10.1093/nar/gni007 PMC546178

[26] Zhao W, Brook MA, Li Y (2008) Design of gold nanoparticle-based colorimetric biosensing assays. Chembiochem 9: 2363–2371. 18821551 10.1002/cbic.200800282

[27] Li H, Yang Y, Wang Y, Li W, Bi L, et al (2010) In situ fabrication of flower-like gold nanoparticles in surfactant-polyoxometalate-hybrid spherical assemblies. Chem Commun (Camb) 46: 3750–3752. 20411186 10.1039/b916797h

[28] Schulz-Dobrick M, Sarathy KV, Jansen M (2005) Surfactant-free synthesis and functionalization of gold nanoparticles. J Am Chem Soc 127: 12816–12817. 16159272 10.1021/ja054734t

[29] Fenger R, Fertitta E, Kirmse H, Thunemann AF, Rademann K (2012) Size dependent catalysis with CTAB-stabilized gold nanoparticles. Phys Chem Chem Phys 14: 9343–9349. 22549475 10.1039/c2cp40792b

[30] Cheng X, Bing T, Liu X, Shangguan D (2009) A label-free fluorescence sensor for probing the interaction of oligonucleotides with target molecules. Anal Chim Acta 633: 97–102. 19110122 10.1016/j.aca.2008.11.031

[31] Liu X, Abbott NL (2010) Characterization of the nanostructure of complexes formed by single- or double-stranded oligonucleotides with a cationic surfactant. J Phys Chem B 114: 15554–15564. 21062067 10.1021/jp107936b

[32] Santhiya D, Dias RS, Shome A, Das PK, Miguel MG, et al (2009) Role of linker groups between hydrophilic and hydrophobic moieties of cationic surfactants on oligonucleotide-surfactant interactions. Langmuir 25: 13770–13775. 19681626 10.1021/la901546t

[33] Zhou S, Liang D, Burger C, Yeh F, Chu B (2004) Nanostructures of complexes formed by calf thymus DNA interacting with cationic surfactants. Biomacromolecules 5: 1256–1261. 15244438 10.1021/bm034524d

[34] Wu Y, Liu L, Zhan S, Wang F, Zhou P (2012) Ultrasensitive aptamer biosensor for arsenic(III) detection in aqueous solution based on surfactant-induced aggregation of gold nanoparticles. Analyst 137: 4171–4178. 22842645 10.1039/c2an35711a

[35] Jeon W, Lee S, Dh M, Ban C (2013) A colorimetric aptasensor for the diagnosis of malaria based on cationic polymers and gold nanoparticles. Anal Biochem 439: 11–16. 23583275 10.1016/j.ab.2013.03.032

[36] Grabar KC, Freeman RG, Hommer MB, Natan MJ (1995) Preparation and Characterization of Au Colloid Monolayers. Analytical Chemistry 67: 735–743.

