# Supplementary material for: Cationic Surfactant-Based Colorimetric Detection of Plasmodium Lactate Dehydrogenase, a Biomarker for Malaria, Using the Specific DNA Aptamer
Source: PLoS One. 2014 Jul 3;9(7):e100847. doi: 10.1371/journal.pone.0100847 (PMC4081113; doi:10.1371/journal.pone.0100847)

**Supporting Information 1**

**Fig. S1. TEM image of the synthesized AuNPs**

The AuNPs were synthesized with a citrate reduction of HAuCl_4_ and their size confirmed using TEM.


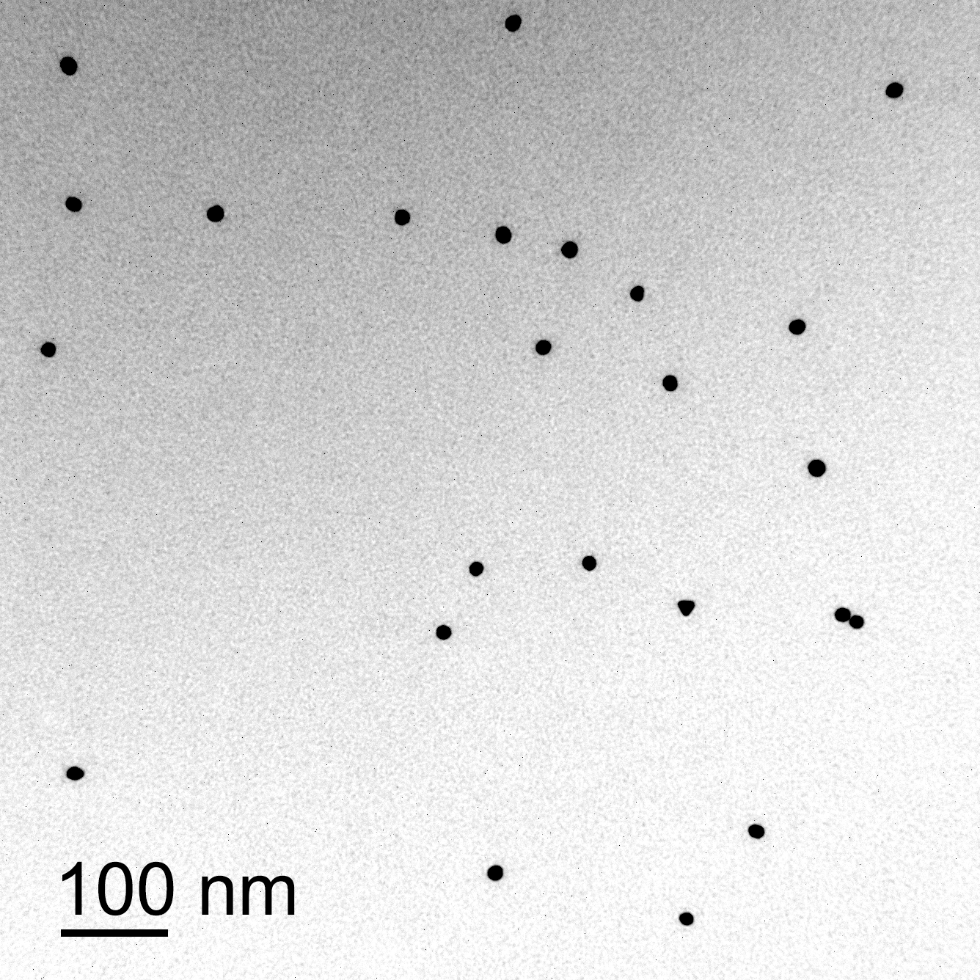

Supplement: Figure S1 — TEM image of the synthesized AuNPs. The AuNPs were synthesized with a citrate reduction of HAuCl4 and their size confirmed using TEM. (DOCX) [file pone.0100847.s001.docx]
