# Supplementary material for: Cationic Surfactant-Based Colorimetric Detection of Plasmodium Lactate Dehydrogenase, a Biomarker for Malaria, Using the Specific DNA Aptamer
Source: PLoS One. 2014 Jul 3;9(7):e100847. doi: 10.1371/journal.pone.0100847 (PMC4081113; doi:10.1371/journal.pone.0100847)

**Supporting Information 2**

**Fig. S2.** **Colorimetric detection of recombinant *Pf*LDH**

(A) The calibration curve of the sensing solutions containing varying concentrations of *Pf*LDH. Points and error bars represents the means and standard deviations, respectively, of three repeated measurements. The inset figures represent the visual color changes of the AuNP solutions. (B) The absorbance spectra for the detection of *Pf*LDH.


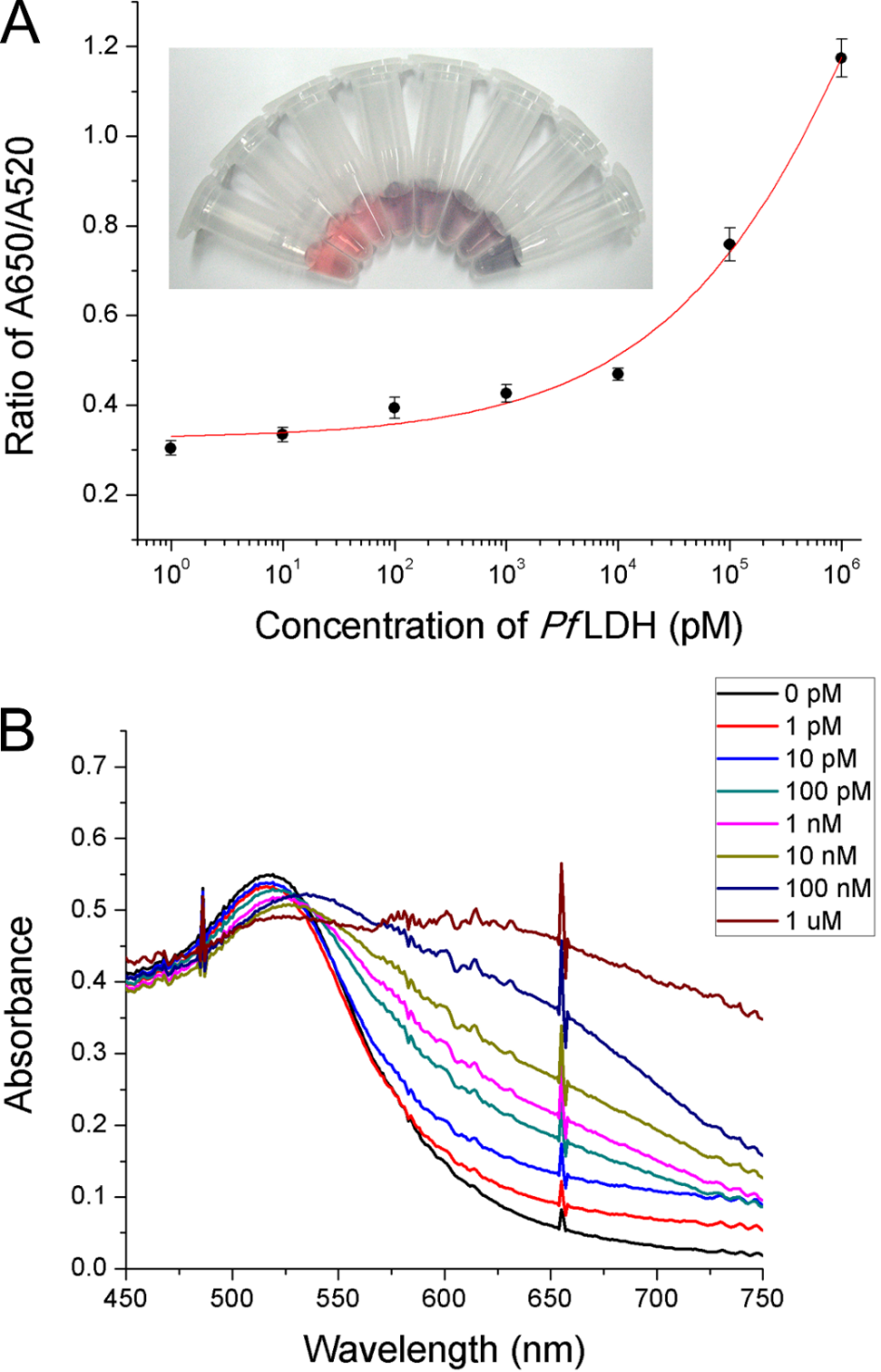

Supplement: Figure S2 — Colorimetric detection of recombinant PfLDH. (A) The calibration curve of the sensing solutions containing varying concentrations of PfLDH. Points and error bars represents the means and standard deviations, respectively, of three repeated measurements. The inset figures represent the visual color changes of the AuNP solutions. (B) The absorbance spectra for the detection of PfLDH. (DOCX) [file pone.0100847.s002.docx]
