# Supplementary material for: Cationic Surfactant-Based Colorimetric Detection of Plasmodium Lactate Dehydrogenase, a Biomarker for Malaria, Using the Specific DNA Aptamer
Source: PLoS One. 2014 Jul 3;9(7):e100847. doi: 10.1371/journal.pone.0100847 (PMC4081113; doi:10.1371/journal.pone.0100847)

**Supporting Information 3**

**Fig. S3. The selectivity of the aptasensor**

The selectivity of the aptasensor for the pLDH proteins. The values of A650/A520 of each competing protein were measured with the same concentration of 1 µM. Error bars represents the standard deviations of three repeated measurements.


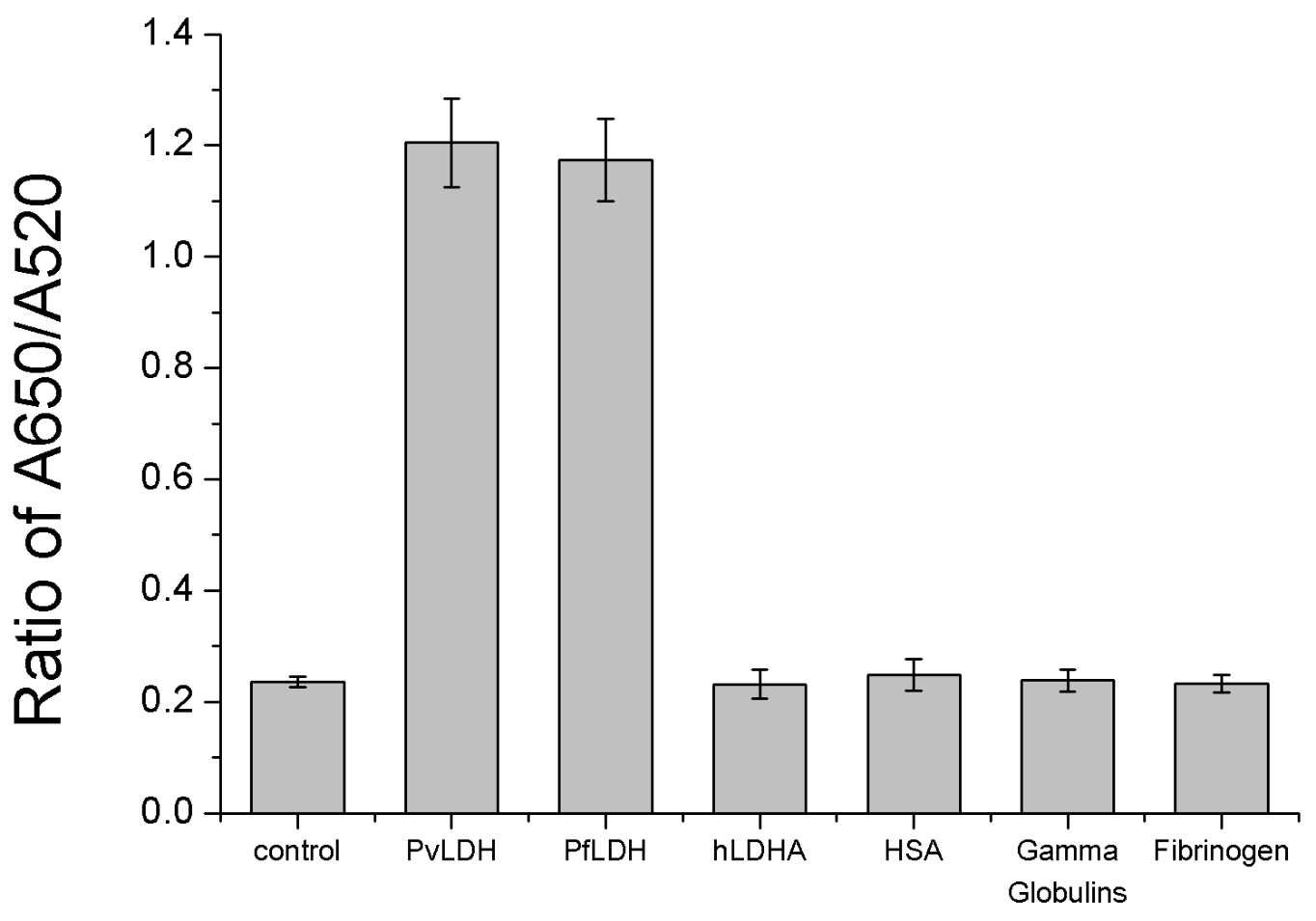

Supplement: Figure S3 — The selectivity of the aptasensor. The selectivity of the aptasensor for the pLDH proteins. The values of A650/A520 of each competing protein were measured with the same concentration of 1 µM. (DOCX) [file pone.0100847.s003.docx]
