# Supplementary material for: Cationic Surfactant-Based Colorimetric Detection of Plasmodium Lactate Dehydrogenase, a Biomarker for Malaria, Using the Specific DNA Aptamer
Source: PLoS One. 2014 Jul 3;9(7):e100847. doi: 10.1371/journal.pone.0100847 (PMC4081113; doi:10.1371/journal.pone.0100847)
Supplement: Figure S4 — Optimization of CTAB concentration for detection in serum samples. The effect of the concentration of CTAB on pLDH detection in serum sample. Points and error bars represents the means and standard deviations, respectively, of three repeated measurements. (DOCX) [file pone.0100847.s004.docx]

**Supporting Information 4**

**Fig. S4. Optimization of CTAB concentration for detection in serum samples**

The effect of the concentration of CTAB on pLDH detection in serum sample. Points and error bars represents the means and standard deviations, respectively, of three repeated measurements.

**
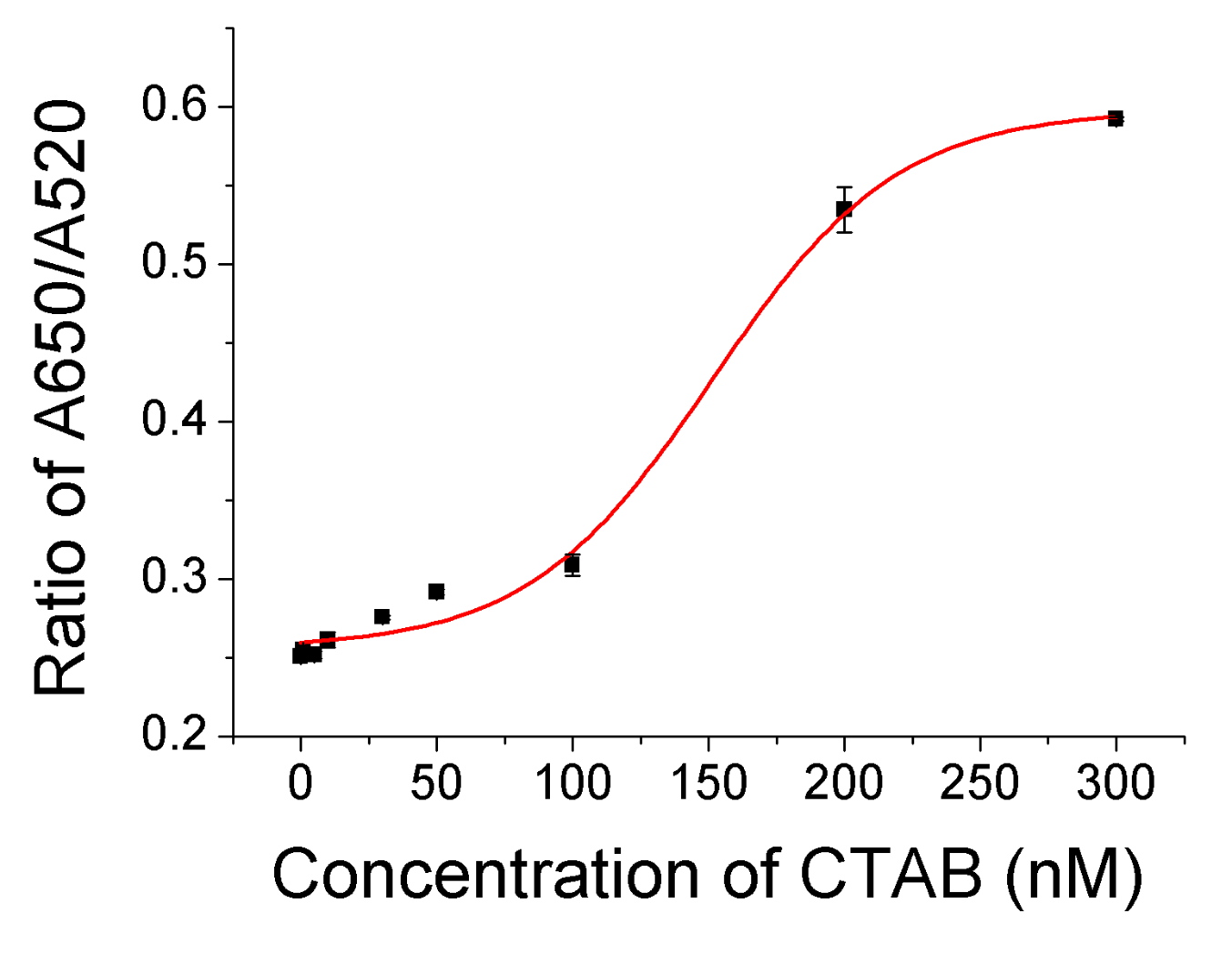
**
