# Supplementary material for: Cationic Surfactant-Based Colorimetric Detection of Plasmodium Lactate Dehydrogenase, a Biomarker for Malaria, Using the Specific DNA Aptamer
Source: PLoS One. 2014 Jul 3;9(7):e100847. doi: 10.1371/journal.pone.0100847 (PMC4081113; doi:10.1371/journal.pone.0100847)

**Supporting Information 5**

**Fig. S5. Detection of *Pf*LDH in the human serum sample**

The calibration curve of the sensing solutions containing varying concentrations of *Pf*LDH in the serum sample. Points and error bars represents the means and standard deviations, respectively, of three repeated measurements.


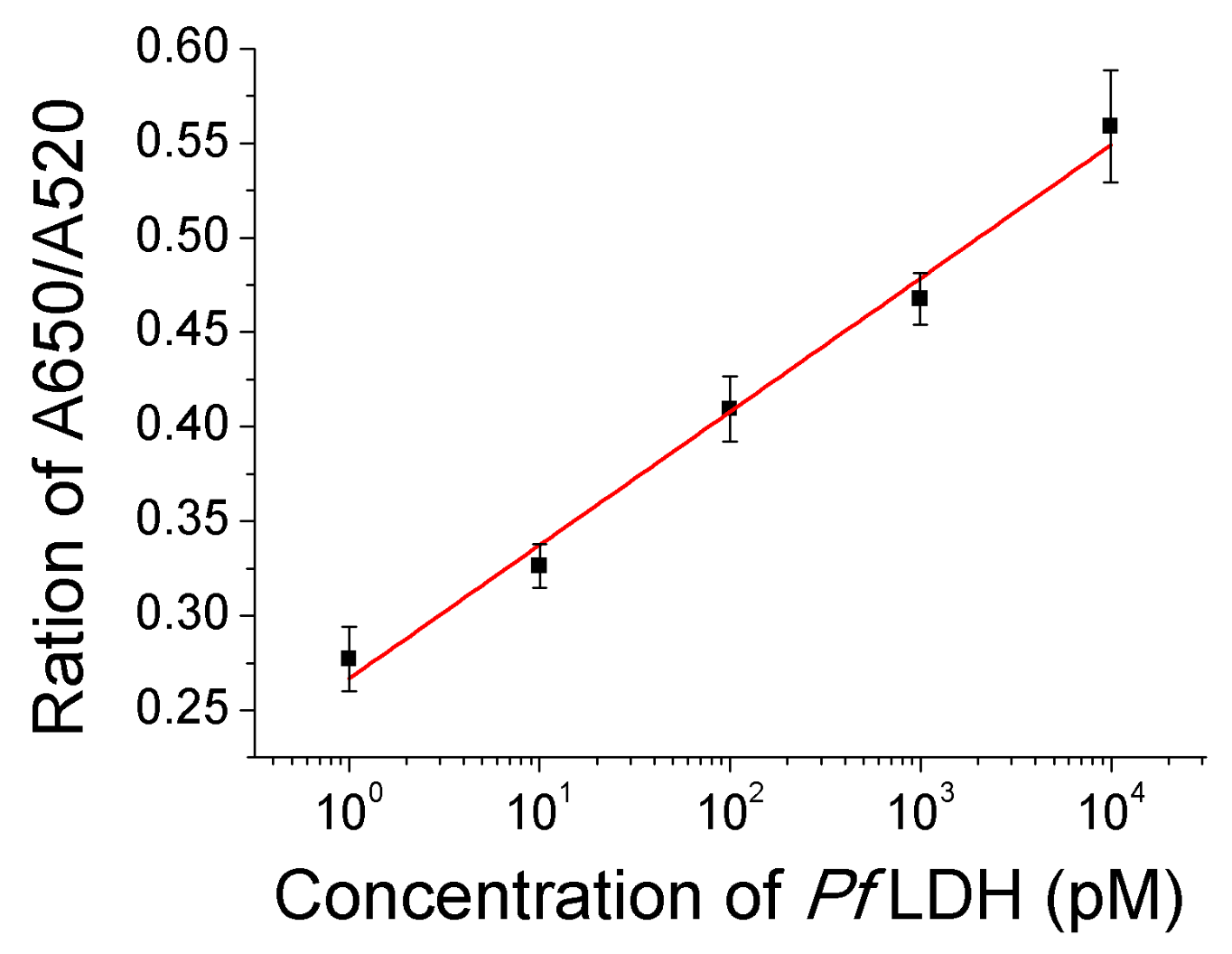

Supplement: Figure S5 — Detection of PfLDH in the human serum sample. The calibration curve of the sensing solutions containing varying concentrations of PfLDH in the serum sample. Points and error bars represents the means and standard deviations, respectively, of three repeated measurements. (DOCX) [file pone.0100847.s005.docx]
